# Early Allograft Dysfunction after liver transplantation– definition, incidence and relevance in a single-centre analysis

**DOI:** 10.1007/s00423-025-03633-8

**Published:** 2025-02-19

**Authors:** Bengt A. Wiemann, Oliver Beetz, Clara A. Weigle, Philipp Tessmer, Simon Störzer, Dennis Kleine-Döpke, Florian W. R. Vondran, Nicolas Richter, Moritz Schmelzle, Felix Oldhafer

**Affiliations:** 1 Department of General, Visceral and Transplant Surgery, Hannover Medical School, Carl-Neuberg-Str. 1, D-30625 Hannover, Germany; 2 Department of General, Visceral, Pediatric and Transplant Surgery, RWTH Aachen University Hospital, Pauwelsstr. 30, D-52074 Aachen, Germany

**Keywords:** Liver transplantation, Early allograft dysfunction, Outcome, Overall survival, Graft survival

## Abstract

**Purpose:**

Early Allograft Dysfunction (EAD) is a serious complication following liver transplantation. With more marginal donors and critical recipients, identifying EAD risk factors and their impact on long-term outcomes is crucial.

**Methods:**

We reviewed all liver transplants performed between 2007 and 2017 at our institution, excluding pediatric recipients, combined thoracic transplants, and retransplants in the same hospital stay. EAD was defined as either: (i) AST/ALT > 2000 IU/l in first 7 postoperative days (POD), (ii) Bilirubin ≥ 10 mg/dl on POD 7, (iii) INR ≥ 1.6 on POD 7.

**Results:**

Of the 621 cases analyzed, the EAD rate was 53.6%. Multivariate analysis identified only donor-dependent variables as independent risk factors for the onset of EAD: donor age (*p* = 0.012), donor serum sodium (*p* = 0.021), cold ischemic time (*p* = 0.007) and graft weight (*p* < 0.001). EAD significantly impaired graft survival (69.2% vs. 86.2% after 1 year; *p* = 0.005) but did not impact long-term patient survival (76.3% vs. 87.6% after 1 year; *p* = 0.162). Of the EAD components, elevated INR proved to be the only reliable predictor of patient mortality. Additionally, an AST/ALT concentration of > 4000 IU/l significantly improved the predictive value of the EAD definition for patient survival (*p* = 0.002).

**Conclusions:**

EAD risk factors are primarily donor-based and significantly impair graft but not patient survival. The high EAD rates and increased use of marginal grafts suggest the need to adjust conventional EAD definitions to optimize graft allocation in the future

## Introduction

Liver transplantation remains to be the standard of care for patients with end stage liver disease. Despite optimization of surgical techniques and perioperative management as well as more personalized immunosuppressive regimens, critical issues remain [1].

The ongoing organ shortage crisis and the increased utilization of extended-criteria donor (ECD) organs correspond with increased risks of biliary complications, organ dysfunction, failure or even loss [2].

Upon engraftment, extended-criteria or marginal organs can enter a state termed as Early Allograft Dysfunction (EAD) with impaired hepatic synthesis leading to significantly inferior short- and long-term outcome of affected recipients [3, 4].

Over the past decades, several definitions of EAD have been published. Among these, the definition by Olthoff et al. published in 2010 is most commonly applied in adult liver transplantation and defines EAD as the presence of one or more of the following criteria: alanine aminotransferase (ALT) or aspartate aminotransferase (AST) > 2000 IU/l within the first seven postoperative days (POD), bilirubin ≥ 10 mg/dl and/or international normalized ratio ≥ 1.6 on POD 7. By applying this definition, the authors identified EAD in 23.2% of the analyzed recipients, together with significantly increased graft loss rates and mortality [4].

Accordingly, prediction of EAD could be key in allocating organs and matching donors and recipients. Olthoff et al. identified donor age and recipient Model for End Stage Liver Disease (MELD) score as prognostic factors in their multivariable model [4]. Other authors, partially applying different definitions of EAD, additionally observed male donors, prolonged donor hospital stay, acidosis, elevated gamma-glutamyl transferase (Gamma-GT), elevated donor body-mass-index (BMI) and donation after cardiac death as relevant prognostic factors for the occurrence of EAD [3, 5–8]. Apart from the mentioned MELD score, risk factors regarding recipients with EAD were identified by various authors and include retransplantation, ventilation or dialysis prior engraftment, prolonged operation time and intraoperative transfusion of packed red blood cells (PRBC), as well as male gender as protective factor [3, 4, 6, 9].

Lastly, graft-associated risk factors, which have been determined in the past, include graft steatosis, elevated graft weight, prolonged cold-ischemia times, and prolonged duration of hepatectomy as well as engraftment (i.e., warm ischemia time) [3, 5, 10–12].

However, given the multifactorial nature a reliable prediction of EAD is still lacking.

Aim of this single center study analyzing liver transplants over the course of ten years in a large liver transplant center in Germany was to evaluate the incidence of EAD in times of organ shortage, the consequences on short- and long-term outcomes as well as the identification of risk factors for the onset of EAD and to evaluate the definitions validity in a country without donation after cardiac death and high levels of ECD donors. These insights could prove helpful for better allocation of grafts and the application of modern preconditioning techniques, such as machine perfusion protocols, in the future.

## Materials and methods

### Study design

We retrospectively analyzed 1035 cases of patients undergoing liver transplantation at the Department of General, Visceral and Transplant Surgery at Hannover Medical School in Hannover, Germany between 1st of January 2007 and 31st of December 2017. Cases were excluded when one of the following criteria was met: recipient age under 18 years, combined cardiac or lung transplantation, living donor liver transplantation and retransplantation in the same hospital stay. Since donation after cardiac death is prohibited by law in Germany, all livers were harvested after brain death of the respective donors. The last date of follow-up was the 22nd of February 2022 resulting in a graft- and patient-follow-up time of at least five years. With the last recorded case being transplanted in 2017, machine perfusion was not used in any cases of this study since it was only introduced to our program in 2021. Standard immunosuppressive regimen at our institution is induction therapy with Basiliximab, followed by calcineurin-inhibitors, Mycophenolate-Mofetil and corticosteroids.

### Definition of variables

We defined EAD as the presence of either: (i) ALT/AST > 2000 IU/l within first 7 PODs, (ii) bilirubin ≥ 10 mg/dl on POD 7 or (iii) INR ≥ 1.6 on POD 7 in accordance with the definition published by Olthoff et al. [4]. Donor hepatectomy time was defined as time between the start of cold perfusion and hepatectomy. Primary aortic anastomosis was defined as at least one vessel being directly anastomosed onto the recipient’s aorta. Anastomoses were defined as “standard” if one vessel of the donor was directly anastomosed to one vessel of the recipient (excluding the aorta). All other anastomoses were defined as “complex vessel reconstructions” (e.g. usage of additional vascular grafts). ECD organs were defined using Eurotransplant criteria when one of the following was met: donor age > 65 years, duration of mechanical ventilation > 7 days, BMI > 30, Bilirubin > 3 mg/dl, AST > 105 IU/l, ALT > 90 IU/l, sodium > 165 mmol/l, hepatic steatosis > 40%.Rejection in the first 90 days was defined as either suspected or confirmed rejection that were treated with corticosteroids.

### Statistical analysis

Statistical analysis was performed using IBM SPSS Statistics Version 28 (SPSS Inc.; IBM Corporation, Armonk, NY, USA). All metric data were analyzed for normal distribution using the Kolmogorov-Smirnov test and, in the case of normal distribution, using Student’s t-test. For the comparison of non-normally distributed metric variables the Mann-Whitney-U test was applied. Categorical variables were analyzed using the Chi^2^ test or Fisher-Exact test. Survival time analyses were performed using Kaplan-Meier analyses, and groups were compared by log-rank and Breslow tests where appropriate. P-values for log-rank and Breslow tests were abbreviated with “P_log_” and “P_Bres_”. Unless otherwise specified log-rank test was used. In this case the estimator calculated by SPSS has been reported as the mean survival estimate. In each case, the significance threshold was set at an α-error of < 0.05. Logistic regression analyses were performed using binary logistic regression. Only variables known before transplantation, with missing values of less than 10% and a p-value of *p* < 0.3 were included in the multivariate regression model to identify risk factors for the onset of EAD. Odds Ratio (OR) was given with 95% confidence interval (CI). Independent risk factors were identified as such when both forward selection and backward elimination proved them as significant. All figures were generated using Graph Pad Prism (version 9.1.0 for Mac, GraphPad Software, La Jolla, CA, USA).

### Ethics

The Ethics Committee of Hannover Medical School has provided guidance for this study under the number “10268_BO_K_2022”. All patients included in the analysis have consented to the use of their data for scientific purposes as it is the general policy at our institution. Personal data were anonymized prior to analysis.

## Results

### Incidence of EAD

After application of the exclusion criteria 631 cases remained. Ten of these cases were further excluded due to either graft failure and retransplantation without the development of EAD (*n* = 1) or patient death without the possibility of developing EAD (*n* = 9), thus resulting in 621 cases. Data regarding donor and recipient demographics as well as surgical details are shown in Table 1. Of the remaining 621 cases, 338 cases (54.4%) developed EAD. The EAD rate ranged from 45.0% in 2010 to 74.4% in 2017. EAD criteria were mainly met by AST/ALT > 2000 IU/l within the first 7 PODs (*n* = 295; 87% of EAD-cases). Most of these transaminase elevations were recorded either directly after transplantation on POD 0 (*n* = 229; 67.7% of cases) or POD 1 (58.8%). Transaminase elevations extending into the 3rd (14.7%) and 7th POD (1.1%) were observed much less frequently. Bilirubin ≥ 10 mg/dl on POD 7 was the second most frequent criteria for EAD recorded in 91 (26.9%) cases. INR ≥ 1.6 on POD 7 was only observed in 13.6% of the included cases.

**Table 1:** Characteristics of all cases (*n* = 631) and after further exclusion (*n* = 10) 621 recipients and donors with (*n* = 338) and without (*n* = 283) the development of EAD compared

Variable			All cases	EAD subgroup	No EAD subgroup		
			*N*_*abs*_ *(N*_*%*_ *) / Mean; Median [Range]*	*N*_*abs*_ *(N*_*%*_ *) / Mean; Median [Range]*	*N*_*abs*_ *(N*_*%*_ *) / Mean; Median [Range]*	*missing value (%)*	*P-value*
Recipient baseline characteristics	Number of cases		631 (100%)	338 (54.4%)	283 (45.6%)		
	Male sex		367 (58%)	195 (57.7%)	167 (59%)	0	0.740
	Age (years)		49.35; 52 [18–73]	49.19; 51 [19–73]	49.63; 52 [18–71]	0	0.652
	Body weight (kg)		76.92; 75 [25–133]	77.09; 76 [25–133]	76.86; 75 [29–131]	0	0.619
	Body height (cm)		173.24; 173 [143–204]	173.23; 172 [143–198]	173.23; 174 [143–204]	0	0.898
	BMI (kg/m^2^)		25.52; 25.14 [12.22–44.98]	25.58; 25.15 [12.22–44.98]	25.51; 25.21 [14.18–41.52]	0	0.747
	Laboratory MELD		22.12; 20 [6–40]	22.12; 19.5 [6–40]	21.89; 21 [6–40]	0	0.755
	Exception MELD		28.84; 29 [20–40]	28.45; 28.5 [20–40]	29.34; 29 [20–40]	331 (52.5%)	0.079
	Underlying disease	Viral hepatitis	158 (25%)	84 (24.9%)	74 (26.1%)	0	0.784
		ASH / NASH	99 (15.7%)	51 (15.1%)	45 (15.9%)		
		Cystic liver disease	45 (7.1%)	22 (6.5%)	22 (7.8%)		
		Metabolic disorder	38 (6%)	24 (7.1%)	14 (4.9%)		
		Cholangitis	103 (16.3%)	51 (15.1%)	50 (17.7%)		
		Cryptogenic cirrhosis	55 (8.7%)	30 (8.9%)	24 (8.5%)		
		Others	133 (21.1%)	76 (22.5%	54 (19.1%)		
	Reason for transplant	Cirrhosis	193 (30.6%)	92 (27.2%)	95 (33.6%)	0	0.225
		Acute liver failure	108 (17.1%)	63 (18.6%)	44 (15.5%)		
		Hepatocellular carcinoma	144 (22.8%)	85 (25.1%)	59 (20.8%)		
		Cholangitis	60 (9.5%)	30 (8.9%)	29 (10.2%)		
		Cystic liver disease	45 (7.1%)	22 (6.5%)	22 (7.8%)		
		Chronic transplant failure	45 (7.1%)	22 (6.5%)	23 (8.1%)		
		Others	36 (5.7%)	24 (7.1%)	11 (3.9%)		
	Previous transplantation		47 (7.4%)	22 (6.5%)	25 (8.8%)	0	0.275
	Time on wait list (days)		365.74; 152 [1-4299]	347.52; 140.5 [1-3462]	389.3; 169 [1-4299]	0	0.163
	High urgency status		83 (13.2%)	50 (14.8%)	32 (11.3%)	0	0.201
Recipient pretransplant clinical status	Home		391 (62%)	214 (63.4%)	173 (61.1%)	0	0.097
	Hospital		115 (18.2%)	53 (15.7%)	62 (21.9%)		
	ICU/IMC		126 (19.8%)	71 (21%)	48 (17%)		
	Diabetes		90 (14.3%)	53 (15.7%)	35 (12.4%)	0	0.238
	On dialysis		116 (18.3%)	64 (18.9%)	47 (16.6%)	0	0.451
	On ventilator		35 (5.5%)	20 (5.9%)	15 (5.3%)	0	0.740
	Laboratory values	Serum sodium (mmol/l)	136.99; 138 [111–164]	136,7; 138 [118–158]	137,34; 138 [111–164]	14 (2.2%)	0.128
		Serum creatinine (µmol/l)	136; 89 [24–810]	136.54; 88 [33–733]	132.97; 89 [24–810]	11 (1.7%)	0.799
		eGFR (ml/min/1.73m^2^)	73.04; 77.68 [4.05-172.91]	72.8; 77.13 [5.44-149.52]	74.20; 80.18 [4.05-172.91]	11 (1.7%)	0.725
		Serum bilirubin (mg/dl)	11.49; 4.70 [0.18–55.06]	11.07; 4.83 [0.18–50.65]	11.70; 4.24 [0.18–55.06]	19 (3%)	0.649
		INR	1.77; 1.43 [0.85-9]	1.74; 1.44 [0.85-9]	1.80; 1.42 [0.85-9]	16 (2.5%)	0.691
		aPTT (sec)	51.3; 42 [14–200]	50.79; 42 [14–160]	52; 42.5 [27–200]	19 (3%)	0.295
Intraoperative detail	Split liver transplantation		41 (6.5%)	19 (5.6%)	20 (7.1%)	0	0.461
	Graft weight (g)		1720.51; 1692 [763–3356]	1834.39; 1826 [807–3356]	1589.73; 1550 [763–3004]	22 (3.5%)	< 0.001
	CIT (minutes)		588.15; 580 [92-1098]	595.53; 586 [240–1098]	578.44; 571 [92-1034]	5 (0.8%)	0.081
	WIT (minutes)		46.35; 44 [20–181]	47.19; 44 [20–181]	45.04; 44 [22–89]	10 (1.6%)	0.184
	PRBC transfusions (n)		8.42; 7 [0–89]	8.97; 7 [0–89]	7.39; 6 [0–35]	7 (1.1%)	0.052
	FFP transfusions (n)		12.69; 10 [0–89]	13.27; 11 [0–89]	11.82; 10 [0–45]	8 (1.3%)	0.123
	TC transfusions (n)		1.36; 1 [0–10]	1.48;1 [0–10]	1.16; 0 [0–7]	7 (1.1%)	0.022
	Intraoperative Resuscitation		22 (3.5%)	18 (5.3%)	2 (0.7%)	4 (0.6%)	0.001
Outcome	Time on ICU/IMC (days)		21.37; 11 [2-243]	24.44; 12 [2-243]	17.97; 9 [2-186]	82 (13%)	< 0.001
	Death on ICU/IMC (days)		82 (13%)	57 (16.8%)	16 (5.7%)	0	< 0.001
	PRBC transfusions first 48 h (n)		4.70; 2 [0–80]	5.72; 2 [0–63]	3.04; 2 [0–31]	18 (2.9%)	< 0.001
	FFP transfusions first 48 h (n)		6.99; 5 [0–65]	8.99; 6 [0–65]	4.3; 3 [0–32]	18 (2.9%)	< 0.001
	TC transfusions first 48 h (n)		1.02; 0 [0–13]	1.31; 0 [0–13]	0.61; 0 [0–10]	18 (2.9%)	< 0.001
	On dialysis during stay		200 (31.7%)	129 (38.2%)	65 (23%)	0	< 0.001
	On dialysis at discharge		27 (4.3%)	17 (5%)	10 (3.5%)	0	0.363
	Wound infection first 30 days		9 (1.4%)	6 (1.8%)	3 (1.1%)	0	0.520
	Suspected or confirmed rejection first 90 days		89 (14.1%)	40 (11.8%)	49 (17.3%)	0	0.054
	Retransplantation in observational period		68 (10.8%)	54 (16%)	13 (4.6%)	0	< 0.001
	Time to retransplantation (days)		369.32; 9.5 [1-4868]	314.83; 7 [1-3204]	623.77; 19 [1-4868]	0	0.065
Donor characteristics	Male sex		356 (56.4%)	212 (62.7%)	140 (49.5%)	0	0.001
	Age (years)		49.94; 51 [10–88]	51.26; 53 [10–88]	48.53; 51 [13–81)	0	0.106
	Body weight (kg)		81.77; 80 [35–230]	85.07; 85 [35–230]	78.11; 78 [40–120]	0	< 0.001
	Body height (cm)		174.88; 175 [140–200]	176.01; 175 [152–200]	173.7; 174 [140–196]	0	0.003
	BMI (kg/m^2^)		26.66; 25.95 [13.84–70.98]	27.38; 26.29 [14.95–70.98]	25.83; 25.25 [13.84–41.52]	0	< 0.001
	Hepatectomy time (min)		45.11; 43 [6-199]	44.66; 43 [6-108]	45.33; 43 [14–199]	54 (8.7%)	0.982
	Time on ICU (d)		5.103; 4 [0.5–40]	5.29; 4 [0.5–40]	4.81; 3 [0.5–32]	2 (0.3%)	0.055
	Time on ventilator (d)		4.92; 4 [0–40]	5.2; 4 [0–27]	4.5; 3 [0–32]	1 (0.1%)	0.013
	Cause of death	Cerebrovascular accident	395 (62.6%)	209 (61.8%)	179 (63.3%)	0	0.941
		Trauma	116 (18.4%)	65 (19.2%)	51 (18%)		
		Anoxic brain damage	71 (11.3%)	39 (11.5%)	30 (10.6%)		
		Others	49 (7.8%)	25 (7.4%)	23 (8.1%)		
	Extended Criteria Donor		347 (55%)	200 (59.2%)	140 (49.5%)	1 (0.2%)	0.018
	Extended Donor Criteria	Serum sodium > 165 (mmol/l)	15 (2.4%)	8 (2.4%)	6 (2.1%)	1 (0.2%)	0.842
		AST > 105 (IU/l)	110 (17.4%)	61 (18%)	46 (16.3%)	1 (0.2%)	0.569
		ALT > 90 (IU/l)	86 (13.6%)	49 (14.5%)	35 (12.4%)	1 (0.2%)	0.450
		Serum bilirubin > 3 (mg/dl)	6 (1%)	4 (1.2%)	2 (0.7%)	1 (0.2%)	0.694
		BMI > 30 (kg/m^2^)	103 (16.3%)	63 (18.6%)	38 (13.4%)	0	0.080
		Donor on ventilator > 7 days	123 (19.5%)	78 (23.1%)	43 (15.2%)	1 (0.2%)	0.014
		Age > 65 (years)	79 (12.5%)	49 (13.3%)	33 (11.7%)	0	0.536
		Liver steatosis > 40%	17 (2.7%)	15 (4.4%)	1 (0.4%)	1 (0.2%)	0.001
		1 criterion fulfilled	198 (31.8%)	103 (30.5%)	91 (32.2%)	1 (0.2%)	0.010
		2 criteria fulfilled	112 (17.7%)	73 (21.6%)	37 (13.1%)		
		3 criteria fulfilled	31 (4.9%)	22 (6.5%)	9 (3.2%)		
		4 criteria fulfilled	6 (1%)	2 (0.6%)	3 (1.1%)		
	Donor laboratory values	Serum sodium (mmol/l)	148.23; 148 [120–199]	149.1; 149 [128–177]	147.19; 147 [120–199]	5 (0.8%)	< 0.001
		Serum creatinine (µmol/l)	101.89; 76 [11.40-898.20]	100.82; 79.6 [11.4–898]	103.33; 72.5 [14.9-804.5]	3 (0.5%)	0.125
		AST (IU/l)	89.43; 47 [5-8204]	100.54; 49 [9-8204]	75.79; 44 [5-892]	12 (1.9%)	0.078
		ALT (IU/l)	74.63; 32 [4-5641]	75.27; 35 [4-5641]	73.52; 28 [4-3384]	6 (1%)	0.013
		Gamma-GT (IU/l)	76.18; 43 [0-775]	88.93; 52 [0-775]	62.74; 35 [5-638]	14 (2.2%)	< 0.001
		Serum bilirubin (mg/dl)	0.70; 0.52 [0.01–6.41]	0.74; 0.55 [0.03–6.41]	0.67; 0.50 [0.017–4.23]	21 (3.3%)	0.355
		INR	1.18; 1.12 [0.81–4.45]	1.15; 1.11 [0.81–3.4]	1.21; 1.14 [0.86–4.45]	45 (7.1%)	0.065

### Risk factors for development of EAD

The final multivariate regression model demonstrated that elevated donor age (OR = 1.019; *p* = 0.012), donor sodium (OR = 1.030; *p* = 0.021), prolonged cold ischemic time (CIT) (OR = 1.002; *p* = 0.007) and elevated graft weight (OR = 1.002; *p* < 0.001) are independent risk factors for the development of EAD (Table 2).

**Table 2:** Univariate and multivariate regression model for the identification of risk factors for the development of EAD

Variable			Univariate Regression			Multivariate Regression		
			OR	CI-95%	*P*-value	OR	CI-95%	*P*-value
Recipient baseline characteristics	Male sex		0.947	[0.688–1.305]	0.740			
	Age (years)		0.997	[0.984–1.010]	0.646			
	Body weight (kg)		1.001	[0.992–1.010]	0.865			
	Body height (cm)		1	[0.984–1.017]	0.997			
	BMI (kg/m^2^)		1.003	[0.971–1.037]	0.857			
	Laboratory MELD		1.002	[0.988–1.015]	0.808			
	Exception MELD		0.955	[0.906–1.006]	0.083			
	Previous transplantation		1.392	[0.767–2.526]	0.277			
	Time on wait list (days)		1	[1–1]	0.382			
	High urgency status		0.734	[0.457–1.181]	0.203			
Recipient pretransplant clinical status	Home		0.911	[0.658–1.262]	0.576			
	Hospital		1.509	[1.005–2.265]	0.047			
	ICU/IMC		0.768	[0.512–1.153]	0.203			
	Diabetes		0.759	[0.479–1.202]	0.239			
	On dialysis		0.853	[0.563–1.291]	0.451			
	On ventilator		0.890	[0.447–1.772]	0.740			
	Laboratory values	Serum sodium (mmol/l)	0.981	[0.953–1.009]	0.173			
		Serum creatinine (µmol/l)	1	[0.999–1.002]	0.719			
		eGFR (ml/min/1.73m^2^)	0.999	[0.995–1.003]	0.653			
		Serum bilirubin (mg/dl)	1	[0.999–1.001]	0.552			
		INR	0.945	[0.812–1.099]	0.461			
		aPTT (sec)	0.998	[0.992–1.004]	0.586			
Intraoperative detail	Split liver transplantation		1.277	[0.667–2.443]	0.460			
	Graft weight (g)		1.001	[1.001–1.002]	< 0.001	1.002	[1.001–1.002]	< 0.001
	CIT (minutes)		1.001	[1-1.002]	0.129	1.002	[1.001–1.004]	0.007
	WIT (minutes)		1.012	[1-1.025]	0.054			
	PRBC transfusions (n)		1.035	[1.009–1.062]	0.008			
	FFP transfusions (n)		1.018	[0.999–1.036]	0.058			
	TC transfusions (n)		1.134	[1.024–1.255]	0.016			
	Intraoperative Resuscitation		0.126	[0.029–0.547]	0.006			
Donor characteristics	Male sex		1.172	[1.247–2.369]	< 0.001			
	Age (years)		1.013	[1.002–1.024]	0.021	1.019	[1.004–1.033]	0.012
	Body weight (kg)		1.027	[1.016–1.038]	< 0.001			
	Body height (cm)		1.027	[1.010–1.045]	0.002			
	BMI (kg/m^2^)		1.075	[1.035–1.116]	< 0.001			
	Hepatectomy time (min)		0.998	[0.987–1.008]	0.629			
	Time on ICU (d)		1.026	[0.989–1.066]	0.175			
	Time on ventilator (d)		1.045	[1.003–1.089]	0.034			
	Extended Criteria Donor		1.426	[1.037–1.962]	0.029			
	Extended Donor Criteria	Serum sodium > 165 (mmol/l)	0.897	[0.307–2.616]	0.842			
		AST > 105 (IU/l)	0.885	[0.581–1.347]	0.569			
		ALT > 90 (IU/l)	1.197	[0.751–1.906]	0.450			
		Serum bilirubin > 3 (mg/dl)	0.596	[0.108–3.281]	0.552			
		BMI > 30 (kg/m^2^)	0.677	[0.437–1.049]	0.081			
		Donor on ventilator > 7 days	1.667	[1.105–2.517]	0.015			
		Age > 65 (years)	0.859	[0.532–1.389]	0.536			
		Liver steatosis > 40%	13.084	[1.717–99.681]	0.013			
	Laboratory values	Serum sodium (mmol/l)	1.030	[1.009–1.051]	0.004	1.030	[1.004–1.056]	0.021
		Serum creatinine (µmol/l)	1	[0.998–1.001]	0.745			
		AST (IU/l)	1	[0.999–1.001]	0.464			
		ALT (IU/l)	1	[0.999–1.001]	0.939			
		Gamma-GT (IU/l)	1.003	[1.001–1.005]	0.002			
		Serum bilirubin (mg/dl)	1.181	[0.899–1.552]	0.231			
		INR	0.441	[0.219–0.887]	0.022			

### Perioperative morbidity after EAD

The development of EAD led to significantly higher rates of postoperative dialysis (38% vs. 23%; *p* < 0.001) and higher blood transfusion requirements of PRBC, fresh frozen plasma (FFP), thrombocyte concentrates (TC); *p* < 0.001), as well as longer Intensive Care Unit (ICU) and Intermediate Care (IMC) stays (24 days vs. 18 days; *p* < 0.001). The occurrence of 90-day mortality was also significantly increased with a rate of 16% (versus 6%; *p* < 0.001). Further data regarding perioperative outcome is shown in Table 1.

### Graft and patient survival

**Fig. 1:**
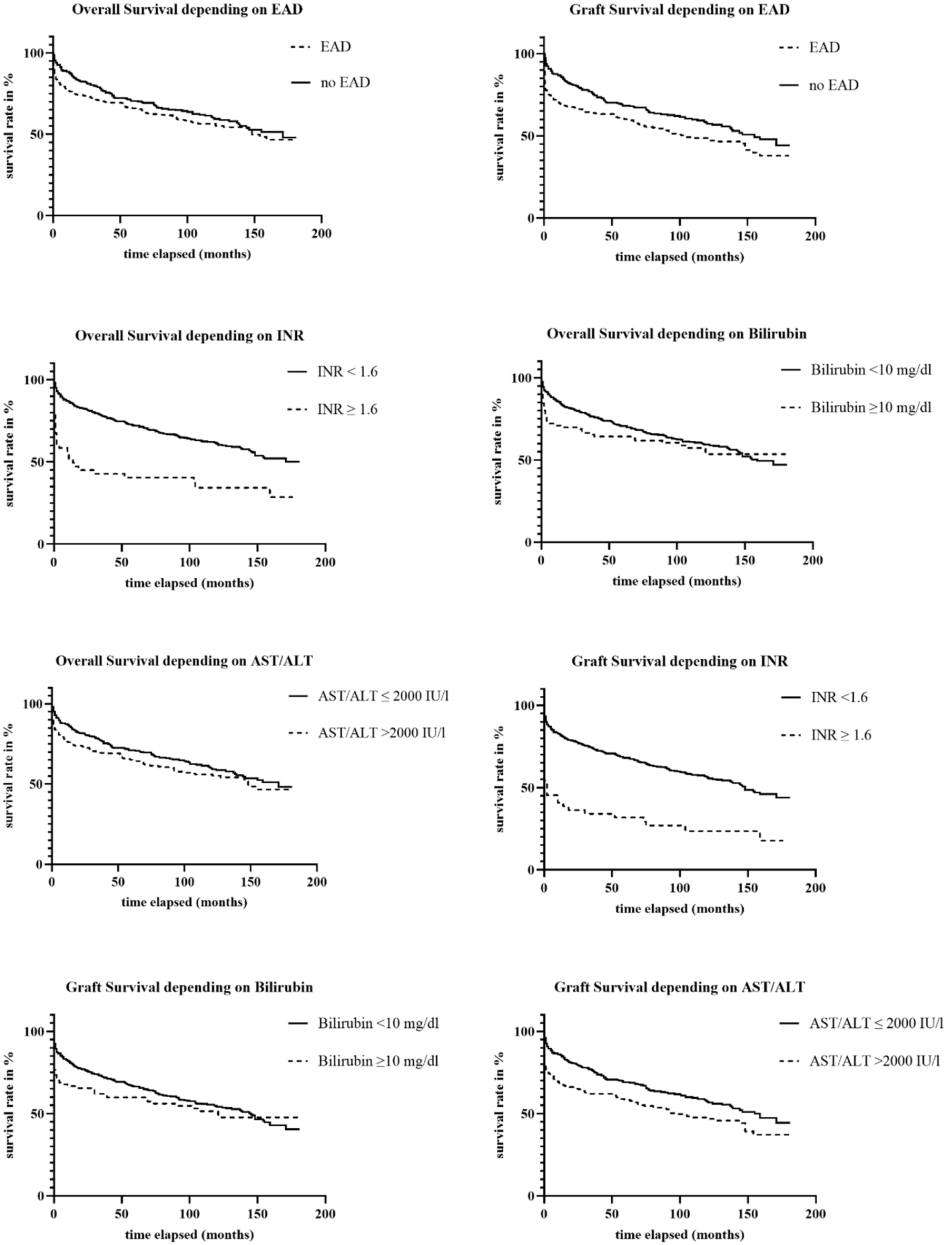
Kaplan-Meier curves for Overall Survival and Graft Survival depending on EAD and the definitions individual components with the x-axis showing the elapsed time in months and the y-axis showing survival rate in percent

Graft survival differed significantly between the two groups with 1-, 3-, 5- and 10-year graft survival rates of 69%, 64%, 60% and 49% in patients who developed EAD compared to all other cases (86%, 75%, 68% and 57%) and a mean graft survival of 98 months (versus 116 months; *p* = 0.005), respectively (Fig. 1).

Overall survival (OS) differed between the two groups, especially in the earlier follow-up period, with 1-, 3-, 5- and 10-year survival rates of 76%, 70%, 66%, and 56% in the EAD recipients (versus 88%, 77%, 71%, and 59% in the non-EAD group) and mean survival estimates of 111 versus 120 months (P_Log_=0.159; P_Bres_=0.038), respectively (Fig. 1). Interestingly, the prognostic value of EAD regarding patient survival depended significantly on the respective EAD criteria met: INR ≥ 1.6 showed a high risk (*p* < 0.001), whereas elevation of bilirubin and transaminases were not significantly associated with inferior overall survival (p-values of *p* = 0.383 and *p* = 0.105, respectively) (Fig. 1).

**Fig. 2:**
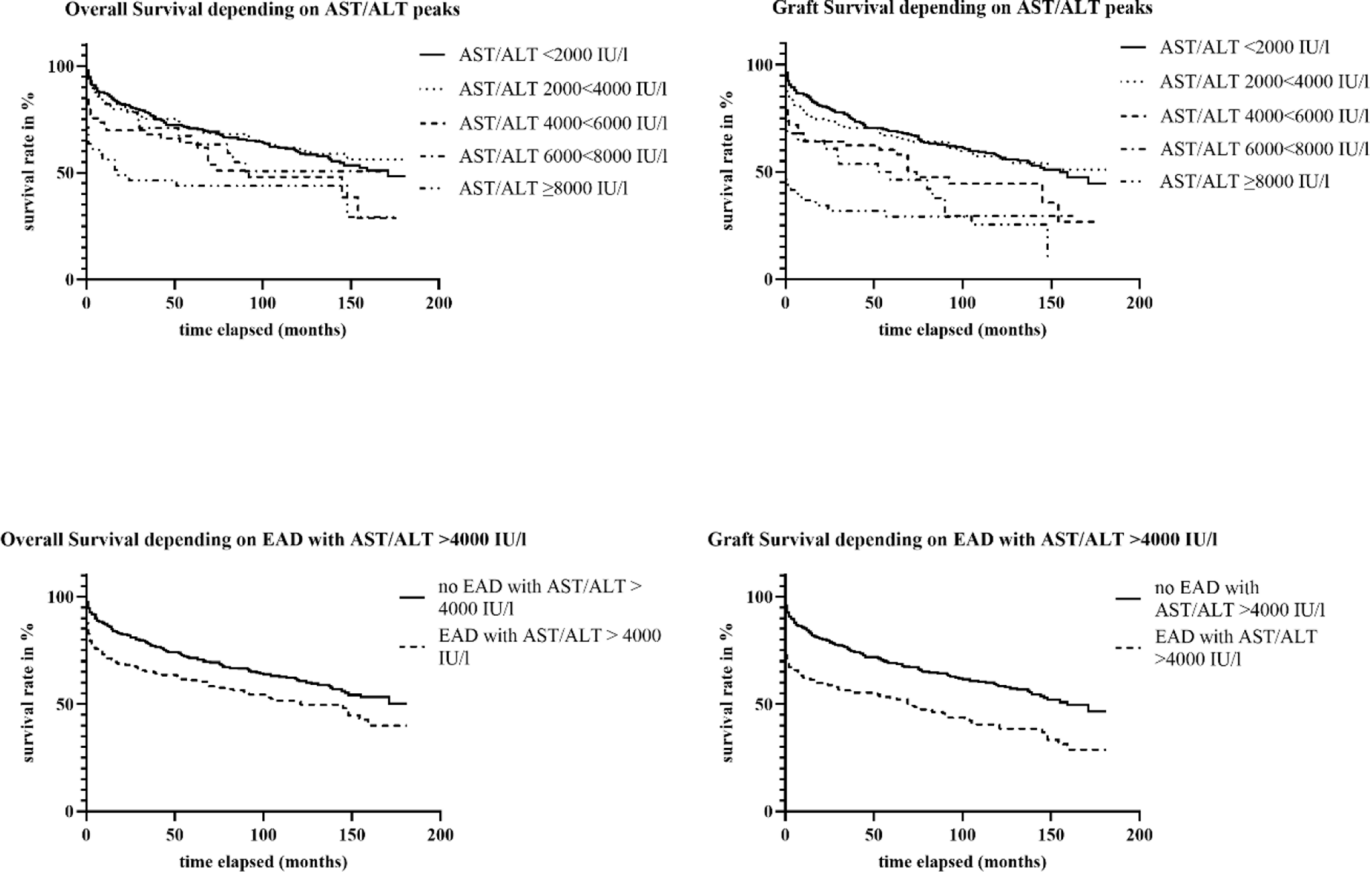
Kaplan-Meier curves for Overall Survival and Graft Survival depending on peak AST/ALT levels and for Overall Survival and Graft Survival depending on EAD with AST/ALT > 4000 IU/l or no EAD with AST/ALT > 4000 IU/l with the x-axis showing the elapsed time in months and the y-axis showing survival rate in percent

Regarding the seemingly low predictive value of elevated transaminases and the high incidence in our collective, we evaluated a range of 2000–4000 IU/l and > 4000 IU/l within the first 7 PODs. Whereas the former showed a comparable mean OS of 122 months (versus 120 months in patients with < 2000 IU/l), the latter showed a significant decline in postoperative OS (mean OS = 94 months in 4000 < 6000 IU/l subgroup; *p* = 0.001). Graft Survival was also significantly impaired in patients with AST/ALT serum concentrations of > 4000 IU/l with a mean OS of 88 months (versus 113 months in the 2000 < 4000 subgroup and 116 in the < 2000 group; *p* < 0.001) (Fig. 2).

Accordingly, a cut-off for AST/ALT of > 4000 IU/l within the EAD-definition could be more appropriate for the definition of EAD regarding the prediction of inferior graft and patient survival (see Fig. 2; *p* < 0.001 for graft survival and *p* = 0.002 for OS). The probability to undergo retransplantation in the further course of follow-up was significantly higher in patients who developed EAD compared to non-EAD recipients with probabilities of 16% and 5%, respectively (*p* < 0.001) (Table 1).

## Discussion

In the context of organ shortage, the careful evaluation, allocation, preservation and reconditioning of potential grafts are of high importance for transplant physicians and surgeons [13]. EAD after liver transplantation has been associated with poor postoperative outcome in publications of the past [3, 6]. However, a reliable definition and stable prognostic markers are still lacking. Furthermore, due to a shift to more marginal donors seen over the past years, previous findings must be reevaluated in large patient collectives [13].

The incidence of EAD in our collective was 54% which is high compared to other publications with Olthoff et al. and others reporting rates from 23.2 to 56% in collectives that, contrary to ours, commonly include donation after cardiac death [4, 14, 15]. We attribute this primarily to the high number of ECD organs (55%) accepted for our patients as well as long cold ischemia times (9.8 h mean; 9.7 h median), high LabMELD values (22 mean; 20 median) and corresponding high frailty and burden of disease (e.g. 18% in need of dialysis pre-transplant) in our recipients, reflective of the profound organ donor shortage in Germany which could lead to the acceptance of organs of poorer quality compared to other European and non-European countries. Hoyer et al. for instance report a mean LabMELD of 18.7 and Lee et al. a mean MELD of 18.4 in their respective EAD-subgroups [3–5, 13, 15–17].

We were able to identify the following variables as risk factors for the onset of EAD: elevated donor age and donor serum sodium, prolonged CIT, and increased graft weight. All these variables are donor-based variables which have been validated in the literature before. Donor age is an established risk factor for graft dysfunction after liver transplantation and has already been identified as relevant in the publication by Olthoff et al. [4, 8, 9]. Hoyer et al. further identified last donor gamma-glutamyl transferase and donor Body Mass Index as risk factors, a trend that was confirmed by Moosburner et al., which differed significantly in our recipients with and without EAD but were not independent prognostic factors [5, 7]. In accordance with our data, Bastos-Neves et al. identified elevated donor serum sodium as significant risk factor [6]. An explanation for these observations was beyond the scope of our analysis. However, Gonzalez et al. hypothesized that the difference in osmolarity between graft and recipient might lead to cellular damage upon engraftment [18]. Prolonged cold ischemic time and elevated graft weight are also well-established risk factors in the literature, coherent to our data [3, 5, 8, 9]. Another often published risk factor was graft steatosis, which was identified for instance by Lee et al. and Hoyer et al. [3, 5]. We were not able to validate this in our data, although we observed statistically significant differences in grafts with > 40% steatosis. Interestingly warm ischemia time, which is also an established risk factor for graft dysfunction, did not differ statistically significant in our collective suffering from EAD. Neither did donor hepatectomy time, which was recently described as a risk factor for poor outcome [10–12].

While many publications explore the association between EAD and mortality, fewer explore the effects on morbidity in the further postoperative course. In our analysis, the development of EAD was associated with significantly longer ICU/IMC stays, higher transfusion requirements and higher rates of post-transplant dialysis.

The association between EAD and inferior graft survival and OS was first reported by Olthoff et al. and while many following publications confirmed the correlation between EAD and inferior graft survival, fewer were able to show a significant difference in OS [4, 7, 19–21]. Ohara et al. did not find significant differences in either, while observing similarly high rates of EAD in their collective and postulated that the prognostic relevance of EAD decreases with the increased utilization of ECD donors. However, in contrast to our patient collective around 30% of donations were performed after cardiac death, where EAD is present in 80% of cases and it has previously been suggested that different mechanisms of EAD occur in donation after cardiac death grafts [14, 17].

We were able to show that EAD significantly influences graft survival and OS especially in the early follow-up time. While Fodor et al. and Mazilescu et al. analyzed the individual components of the EAD definition and were able to show that elevation of AST is a weak and of INR and bilirubin are strong predictors of mortality, this was only partially in line with our observations [17, 22]. Partially in accordance with Figiel et al., who refer to INR as the strongest predictor of early graft loss, INR seemed to be the only parameter as part of the EAD definition that predicted long-term OS accurately while elevation of transaminases and bilirubin did not [23]. Furthermore, the latter was the only parameter which was not associated with inferior short-term OS.

Regarding the evident lack of discriminatory power of elevated peak transaminase levels according to the conventional EAD definition in our high-risk donor-recipient-pool, we chose to evaluate a higher cut-off of > 4000 IU/l, which showed a highly significant association with inferior graft survival and OS and highlighted the importance of elevating the cut-off for transaminase levels in a high morbidity recipient population in combination with a high-risk donor pool. This exact cut-off has not been proposed in the literature before, although Figiel et al. identified a cut-off of AST close to 4000 IU/l as ideal in a recent study exploring the predictive value of transaminase activities towards 90-day graft loss after liver transplantation [24]. Additionally, the relationship between peak transaminase levels and mortality after liver resections has been thoroughly explored [25]. Additionally, Pareja et al. used peak transaminase levels for their “MEAF Score”, a continuous score to grade early transplant dysfunction and Salvalaggio et al. defined “moderate and severe EAD” in the presence of peak transaminase levels of > 3000 IU/l [26, 27].

In summary, EAD is associated with increased morbidity and inferior graft survival, as well as inferior short-term OS. Thus, it is of utmost importance to reduce the rate of EAD after liver transplantation. Machine perfusion, as a novel technique to ameliorate ischemic injury to the liver graft prior engraftment, has the potential to improve the outcome after liver transplantation in this regard [28–30]. However, clear consensus about which grafts should routinely receive machine perfusion is still lacking and we hope to contribute to the current discussion by pointing out the importance of donor-based risk factors [31]. Furthermore, we were able to show that several published risk factors such as warm ischemic and hepatectomy time, but most importantly recipient-related risk factors such as MELD score had no impact on the development of EAD in a high-risk donor-recipient-constellation [3, 4, 6, 11, 12].

Our study has several limitations, inherent in its retrospective and single-center design and additionally the lack of inclusion of donation after cardiac death grafts.

## Conclusion

EAD risk factors are primarily donor-based and significantly impair graft but not patient survival. High EAD rates and increased use of marginal grafts in high-risk recipients suggest the need to adjust conventional EAD definitions by raising the threshold for transaminase elevations to optimize graft allocation and postoperative risk assessment in the future.

## Data Availability

No datasets were generated or analysed during the current study.
